# Programmed Bending Reveals Dynamic Mechanochemical Coupling in Supported Lipid Bilayers

**DOI:** 10.1371/journal.pone.0028517

**Published:** 2011-12-22

**Authors:** Sean F. Gilmore, Harika Nanduri, Atul N. Parikh

**Affiliations:** 1 Department of Applied Science, University of California Davis, Davis, California, United States of America; 2 Department of Biomedical Engineering, University of California Davis, Davis, California, United States of America; 3 Department of Chemical Engineering and Materials Science, University of California Davis, Davis, California, United States of America; Dalhousie University, Canada

## Abstract

In living cells, mechanochemical coupling represents a dynamic means by which membrane components are spatially organized. An extra-ordinary example of such coupling involves curvature-dependent polar localization of chemically-distinct lipid domains at bacterial poles, which also undergo dramatic reequilibration upon subtle changes in their interfacial environment such as during sporulation. Here, we demonstrate that such interfacially-triggered mechanochemical coupling can be recapitulated *in vitro* by simultaneous, real-time introduction of mechanically-generated periodic curvatures and attendant strain-induced lateral forces in lipid bilayers supported on elastomeric substrates. In particular, we show that real-time wrinkling of the elastomeric substrate prompts a dynamic domain reorganization within the adhering bilayer, producing large, oriented liquid-ordered domains in regions of low curvature. Our results suggest a mechanism in which interfacial forces generated during surface wrinkling and the topographical deformation of the bilayer combine to facilitate dynamic reequilibration prompting the observed domain reorganization. We anticipate this curvature-generating model system will prove to be a simple and versatile tool for a broad range of studies of curvature-dependent dynamic reorganizations in membranes that are constrained by the interfacial elastic and dynamic frameworks such as the cell wall, glycocalyx, and cytoskeleton.

## Introduction

An emerging tenet in membrane biophysics is that an interplay between compositional heterogeneity and membrane mechanics holds the key to linking organization with functions. [1] Lipids in biological membranes are not homogeneously dispersed: non-random co-distributions of lipids and proteins, spatial gradients of lipid concentrations, and compositionally differentiated domains are pervasive. While intermolecular interactions-in conjunction with extra-membrane constraints-provide a thermodynamic rationale for understanding domain organization of membranes, [2] a growing body of evidence suggest that the functional domains are often not a passive consequence of membrane equilibration. [3], [4] Rather, domains form, reorganize, and break-up as an active response to a host of physical and chemical perturbations, spatially and temporally organizing lipids (and associated membrane proteins), thereby producing functional hot-spots for signaling and trafficking, within the membrane milieu. [5] The cumulated weight of these studies support the overarching notion that dynamic generation of domains and their mesoscale patterning might be the key organizing principle for membrane functions. [4]

Functional membrane heterogeneities are often linked to membrane mechanics by curvature and dynamics. [6], [7] Curvatures in cellular membranes are modulated by a variety of intrinsic and extrinsic pathways. These include insertion (or generation) of curvature-sensitive lipids and membrane proteins, surface scaffolding by cytoskeletal (re)polymerization, intra- and extracellular forces due to osmotic gradients in the aqueous environment, protein-binding, and motor protein activity. [6] In all of these cases, curvatures elicit spatial and temporal heterogeneity in lipids, which also organize membrane processes.

A dramatic example of such curvature-composition coupling is the polar localization and curvature-dependent lipid targeting, such as in bacterial cells. Here, microscopic domains of cardiolipin (a phospholipid with high, negative curvature) (C = 0.1 nm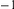), localize at the curved poles. [8] A recent equilibrium description based on free energy consideration suggests that such localization is not determined by molecular curvature alone, but domains large enough to overcome entropic penalties are required to drive such heterogeneity. [9] Indeed, a delicate balance of competing interactions produce conditions for cardiolipin localization. Specifically, local short-range attractive interactions leading to phase-separation facilitate domain formation, which is opposed by the long-range repulsive elastic interactions due to the mismatch between domain curvature and that imposed by the cell-wall. Furthermore, the strength of this curvature-composition coupling can be overcome by intracellular forces. By modulating the balance of these competing interactions, the repartitioning of the cardiolipin domains occurs at the septal plane due to changes in osmotic pressure, which reduces long-range elastic repulsions. Because these mechanochemical couplings are purely physical and/or chemical in nature, they can be recapitulated *in vitro*. Indeed, protein-free GUVs or planar double lipid bilayers reproduce similar mechanochemical coupling under stationary equilibrium conditions. [10], [11]

However, curvatures in biological membranes are rarely static. Mechanochemical mechanisms (see above) dynamically generate and modulate curvatures in living cells at all relevant length scales, which give rise to a dynamic reorganizations of membrane domains. Model systems that recapitulate dynamic curvature-composition coupling, to our knowledge, have been limited to GUVs, where only a restricted range of curvatures can be reliably generated and domain preferences analyzed. A prerequisite for dynamically reconstituting curvature-preference of membrane domains using supported membrane configurations is the ability to modulate its equilibrium properties in real-time.

**Figure 1:**
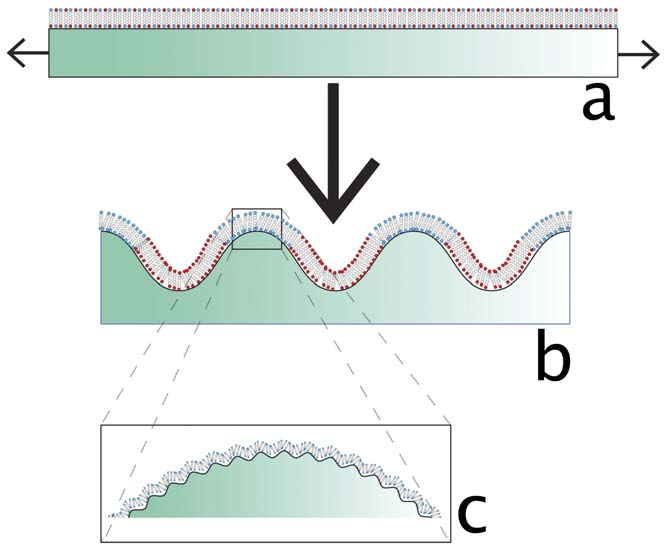
Cartoon of dynamic wrinkling with a bilayer. A cartoon showing a uniform lipid bilayer on a flat and stretched PDMS elastomer a. b, real-time surface wrinkling of the elastomer induces curvature dependent molecular and domain redistribution. c, an inset at the bottom depicts nanoscale topography.

In the work reported here, we introduce a novel model membrane configuration in which curvatures are presented to pre-equilibrated lipid bilayers on command. To achieve this goal, we use a unique property of poly(dimethyl)siloxane (PDMS) elastomers. Specifically, we exploit the observation that stretched, surface-oxidized PDMS instantaneously produces a nested hierarchy of surface curvatures upon release. [12] The first range of curvature has a wavelength of 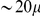
m, and upon these curves are 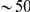 nm wrinkles. The wavelength and amplitude of these features can be controlled by varying the amount by which the PDMS is stretched and the length of UVO exposure, though the dimensions of the two curvatures are related and conform to these relative magnitudes. This nested topography is generated by the mismatch of the Young's modulus of the glassy, silica-enriched oxidized surface and the underlying elastomeric bulk. We have previously shown that simple vesicle fusion onto this hydrophillic silica-like surface produces single lipid bilayers. [13] We now demonstrate that these properties can be combined with real-time substrate restructuring to introduce ranges of one- and two-dimensional curvatures to planar lipid bilayers *in situ* prompting their re-equilibration and attendant domain (re)organization (Figure 1).

## Results

**Figure 2:**
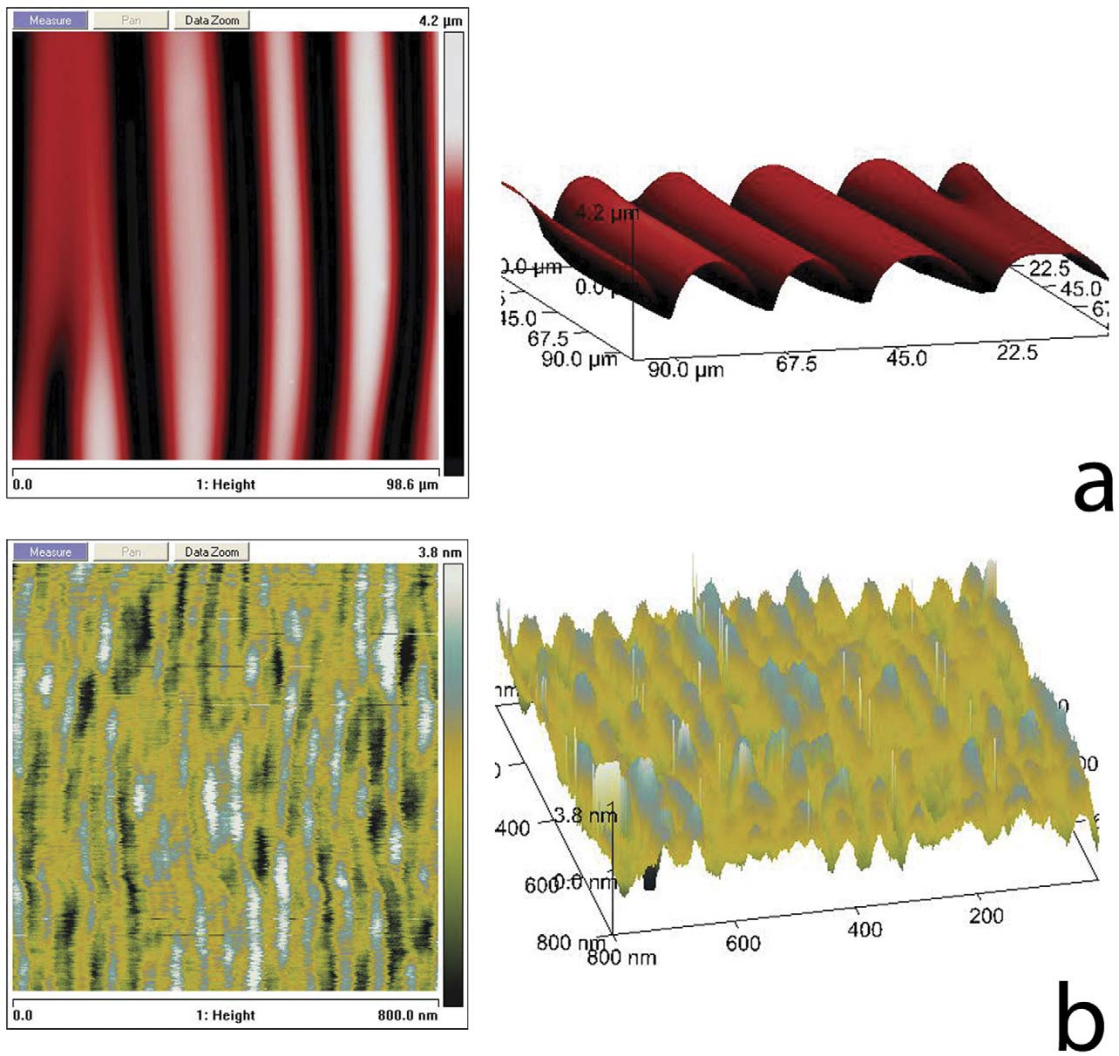
AFM data of wrinkled PDMS. a and b, AFM micrographs of a buckled PDMS substrate, revealing micro- and nano-scale surface corrugations.

To prompt substrate-directed dynamic reequilibration in PDMS supported lipid bilayers, we begin with the fusion of small unilamellar vesicles (SUVs) onto stretched, hydrophilic PDMS elastomers. First, a flat piece of PDMS is uniaxially stretched by 33% of its length, and its surface is exposed to ozone-generating, short-wavelength ultraviolet (UVO) radiation (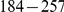 nm) for approximately 30 minutes. The UVO treatment is known to oxidatively remove backbone methyl groups from PDMS molecules and promote cross-linking of newly formed silanol groups in a depth-wise self-limiting manner. This produces a stiff, 5 nm hydrophilic silica-like skin atop the elastic PDMS foundation. The still-stretched PDMS is then incubated with a buffered aqueous solution of SUVs consisting of putative raft-forming mixtures of variable molar proportions of cholesterol (CH), sphingomyelin (SM), and DOPC. These and related ternary lipid mixtures have been extensively studied in recent years and their phase diagrams well-characterized in terms of co-existing liquid-disordered (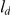) and cholesterol-enriched liquid-ordered (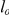) or raft phases, most notably for the giant unilamellar vesicle (GUV) configuration. [14] To enable fluorescence visualization, the lipid mixtures were doped with 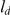-partitioning 1 mol% Texas-red conjugated DHPE lipid, and in selected experiments, raft-partitioning monosialoganglioside (GM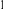) is also used. Subsequent release of the mechanical stretch under the aqueous environment instantaneously remodels the elastomeric substrate producing a corrugated topography. Imaging the substrate surface using optical and scanning probe microscopy reveals the appearance of a periodic pattern of hierarchy of nested one-dimensional curvatures spanning nanometer (approximately 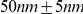) to micrometer (approximately 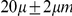) scale in a direction perpendicular to that of the initial stretch (Fig. 2 a and b). These observations are in good conformity with similar findings reported previously. [12]

**Figure 3:**
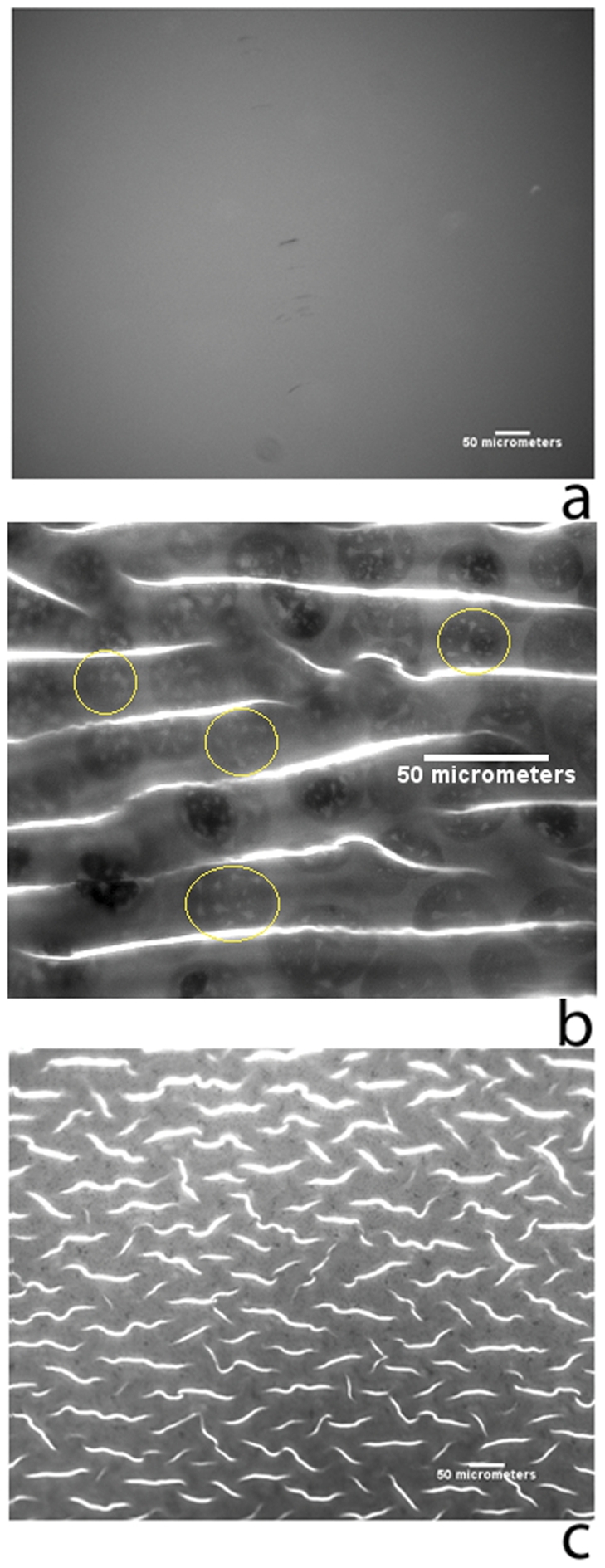
Epifluorescence images of lipid bilayers (1% TR-DHPE, 0.5% GM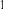, 10% CH, 45% DOPC, 43.5% Egg-SM). (a), a in image of a bilayer on PDMS prior to wrinkling, 10×. (b), an image of the same bilayer in (a) after wrinkling, 20×. (c), an image of a bilayer that was applied to pre-wrinkled PDMS, 10×. Large, oval-shaped domains are visible in (b) and some have been circled in yellow for clarity. Smaller, 1–2 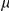
m domains are visible in (c). All scale bars 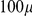
m. The contrast of panel b has been adjusted. The bright lines seen in (b) and (c) are optical artifacts.

Wide-area epifluorescence images provide evidence for substrate-directed reequilibration of the fluorescently-doped lipid bilayer. Images of samples before release of the PDMS reveals bilayer homogeneity at the micron scale (Fig. 3 **a**. After release, (Fig. 3 **b**) we observe a remarkable domain pattern characterized by the presence of a large number of oval shaped domains devoid of fluorescence. The size of the dark domains in the Texas Red channel range from less than 10 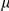
m, to almost 30 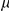
m in major diameter, with their minor axis approaching the micrometer-scale wavelength of the corrugated PDMS. A closer examination of the probe depleted domains reveals an internal structure; nested domains that contain dye appear to be a common feature of these larger, dye-excluding domains. These are reminiscent of those reported for phase-separating lipid monolayers at an air-water interface. [15] Because Texas-Red partitions preferentially in the fluid (or liquid-disordered, 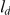) phase, [16] we tentatively ascribe the probe-depleted oval domains to cholesterol-enriched liquid-ordered (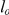) phase of the bilayer.

A closer examination reveals that domain distribution across the sample surface is not random. The oval domains are almost exclusively organized in regions of low curvature, with their major axes aligned preferentially along the directions of the striations of the buckled topography. Note that the bright striations we observe are not specific to phase-separating bilayers, seen routinely also in monocomponent uniform lipid bilayers possibly originating from the optical artifacts associated with the curved substrate topology. This curvature-dependent domain organization parallels previous observations in which liquid-ordered domains were seen to organize - albeit in double bilayers - in regions of low curvature. [11].

To investigate if buckled topography of the substrate is solely responsible for the observed domain morphology, we performed control experiments using substrates that had been buckled prior to bilayer deposition (Fig. 3 **c**). The epifluorescence image shown in Fig. 3 **c** reveals small (1–2 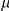
m) features devoid of fluorescence uniformly distributed across the sample surface. Appearance of these features is consistent with the formation of (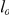) phase domains on supported substrates. Comparing these domains with curvature-dependent organization of larger domains evident in Fig. 3 **b**, we infer that the presentation of curved topology alone is not sufficient to produce curvature-dependent domain localization in single bilayers.

**Figure 4:**
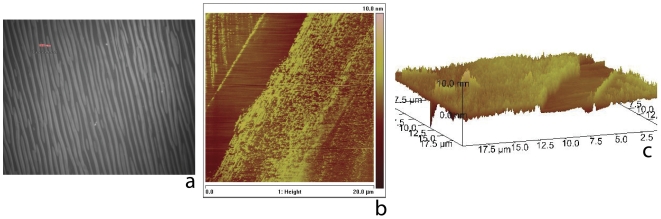
Transfer of bilayer by micro-contact printing from corrugated PDMS elastomers onto silicon substrates. (a) Epifluorescence image of PDMS substrate following micro-contact printing. The image reveals a substantial loss of fluorescence from the PDMS hills, which come in contact with the silicon substrate. (b,c) Atomic force microscopy images (2D rendition, Fig. 4 **b** and 3D rendition, Fig. 4 **c**). The images indicate transferred height of the lipid phase of 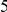 nm confirming the presence of single bilayer onto the parent PDMS. Note also that nanoscale features of the hill region (see text) are reproduced in the transfer process.

To confirm that the lipid phase obtained on PDMS via vesicle fusion is indeed a single bilayer and that the substrate buckling preserves the bilayer motif, we used microcontact printing to transfer the lipid phase from the wrinkled PDMS surface to a clean silicon wafer. [17] Fluorescence images after printing show a significantly diminished fluorescence on the “hills” of the PDMS suggesting near-complete transfer of the lipid bilayer. Quantitative transfer of approximately 5 nanometer thick lipid bilayer from conformal regions of PDMS to silicon is established by a combined application of fluorescence, and AFM measurements (Fig. 4) confirming that single bilayers form on stretched PDMS elastomers and that they retain their bilayer motif upon buckling (Fig. 4 **b** and **c**. It is notable that portions of the bilayer on nanoscale features do not transfer, as evident in Fig. 4 **b** and **c**, accounting for the reduced, but not completely removed fluorescence from the parent PDMS substrate. This lends additional support to the notion that the bilayer adhering to PDMS closely follows the substrate topography, including the nanoscale corrugations.

**Figure 5:**
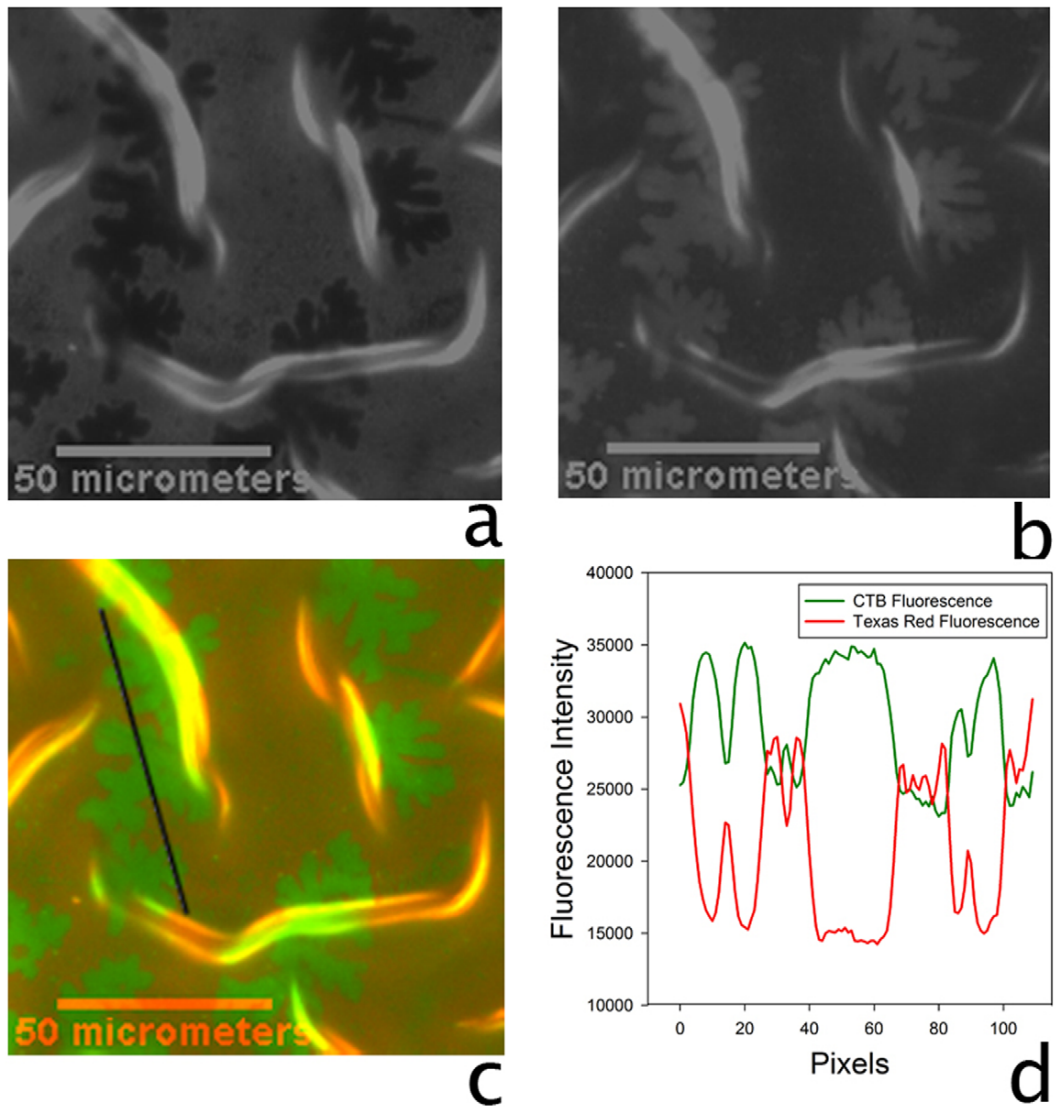
Epiflourescence images showing fluorescent cholera toxin bound to the bilayer. Constrained re-equilibration of a bilayer consisting of TR-DHPE/GM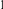/CH/DOPC/Egg-SM (1/0.5/5/74/19.5) on a dynamically buckled PDMS substrate. Fluorescence images taken after FITC-tagged cholera toxin subunit B was applied to a bilayer on PDMS. a, the Texas Red channel; b, the FITC channel; c, the combined fluorescence channel. Scale bar 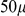
m. Panel d compares the linescan of the fluorescence intensities shown in panel c.

To further verify that the probe-depleted oval domains are indeed 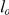 phase domains within the contiguous bilayer, we incorporate GM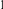, a raft-partitioning molecule, [18] into our bilayers. Binding of GM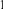 by its fluorescently labeled cholera toxin partner (FITC-labled B5 subunits, CTB) then provides a positive identification of the 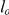 domains. [19] Fig. 5 **a** reveals dendritic, probe-depleted domains in the Texas Red channel. Note that the changed morphology is seen reproducibly in regions of samples with two-dimensional curvature. Fig. 5 **b** shows a complementary fluorescence in the FITC channel in regions that were probe-depleted in the Texas Red channel. The composite fluorescence image reveals green domains (Fig. 5 **c**), which corresponded to the initial probe-depleted domains, in the red surroundings assigned to the 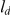 phase. A comparison of linescans from Fig. 5 **a** and **b** confirm the complementary fluorescence (Fig. 5 **d**). The formation of these digitated domains appears unusual since the shapes of liquid ordered domains is typically round, determined by surface tension. We speculate that that domain morphology is not determined by line tension alone; Rather it appears to be strongly influenced by the the interplay between line tension and different curvatures imposed through the two-dimensionaly wrinkled substrate seen in Fig. 5.

A prerequisite for the curvature-dependent domain reorganizations such as observed here is the long-range translational mobility of lipids within the bilayer media. To determine how fluidity is influenced by the topology in our PDMS supported bilayers, we adapted a simple microscopy-based, two-dimensional (2D) fluorescence recovery after photobleaching (FRAP) experiment. [20] We performed FRAP experiments for three distinct types of samples: raft-forming single lipid bilayers on (1) flat, unwrinkled PDMS; (2) dynamically wrinkled PDMS; and (3) PDMS that was pre-wrinkled. We find that the raft-forming bilayers on PDMS, before the wrinkling occurs, exhibit substantial long-range fluidity as measured by the probe diffusion constants (approximately 1 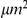/sec at 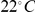) (Figure S1 **a** and **b**). In sharp contrast, the buckled membrane morphology derived by real-time remodeling of the PDMS substrate, the bilayers essentially lose their long-range fluidity exhibiting gel-phase like behavior (at the same temperature). Increasing the temperature of the sample to 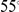
C did not result in any measurable change in fluidity, and no recovery was observed before the sample began to substantially photobleach. Thus, we are not able to accurately measure the significantly reduced fluidity in these samples. A FRAP experiment was repeated on the same raft mixture on pre-buckled PDMS, and this surprisingly showed fluorescence recovery indicating long range fluidity (Figure S1, panels **c** and **d**) (approximately 0.5 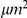/sec at 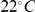). Repeating the dynamic wrinkling experiment with a pure DOPC (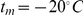) and dye mixture revealed that the lateral diffusion was not arrested. This indicates that the arrest of fluidity is a result of the dynamic wrinkling process and the ternary mixture working in tandem. We speculate that the heterogeneity, which results from the dynamic wrinkling, gives rise to the loss of of long range fluidity.

Taken together, results presented above indicate that real-time topographical buckling of the substrate induces gross morphological reorganization and an attendant fluidity transition in the adherent supported lipid bilayer. For the raft-forming mixtures studied here, the reorganization results in the formation of microscopic 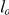 domains, which also organize and orient preferentially along the regions of low substrate curvature. These morphological reorganizations are attended by a sudden arrest of long-range translational mobility of probe molecules within the bilayer environment.

## Discussion

Several factors must be taken into account to understand the type of real-time mechanochemical coupling evident in the results presented above. These are discussed in turn below.

First, the rapid buckling of the PDMS elastomer changes the geometry of the bilayer-surface adhesion energy while simultaneously introducing bending energy. Tension energy can be neglected because the net surface area in our system does not appear to change upon buckling, as the glassy skin strongly resists compression. In order for the membrane to follow the incipient substrate topography, the energy gain due to adhesion must exceed the energy cost of bending the bilayer. The bending energy per unit area is given by the Helfrich equation (Eqn. 1).
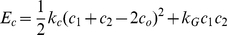
(1)Where 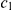 and 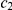 are principle radii of curvature for the bent bilayer and 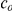 is the average curvature of the constituent lipids. 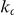 and 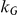 refer to the bending and Gaussian moduli of the bilayer, respectively. The majority of lipids used in the bilayers in our experiments are cylindrical (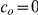). For a membrane such as this, bent over 1D topography (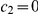), the Helfrich energy expression simplifies to 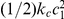. Using an approximate value of 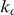 of 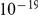
J, [21] and estimating the tightest radius of curvature as 25 nanometers, one can estimate the Helfrich energy at 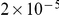
J/m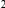. Larger microscopic curvatures, (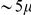
m) generate much smaller energetic contributions (approximately 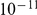 J/m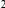). Experimentally reported values of the adhesion energy per unit area between the lipid bilayer and a silica-like substrate range between 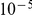 to 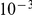 J/m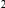. [22] Since the adhesion energy is approximately equal to or greater than the bending energy, the energy imparted into the bilayer is likely insufficient to cause it to separate or unbind from the substrate. Thus, the membrane remains “epitaxially” bound to the emergent topography, which is consistent with the results reported here.

Second, the most prominent consequence of the new bilayer topography is the observed long-range positional alignment of 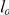 phase domains in the regions of the lowest curvature. This mechanically triggered domain organization is driven by the free energy of the system, which may be regarded as at thermal equilibrium on the diffusion time scale of the bilayer. The differential lowering of bending energy penalty for the organization of 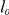 domains in regions of specific curvature must overcome the thermally-excited entropic randomization. In other words, 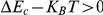. As a first approximation, 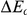 corresponds to the difference in bending energy between the localization of the 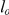 and the 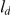 phase at the regions of high curvature. Using the Helfrich bending description, as above, we obtain 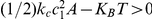. Previous experimental results relating 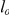 and 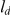 phase moduli estimate that 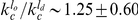. [10] Based on this and assuming 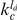 to be approximately 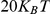, [23] we find that for all radii of curvature below 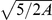, positional alignment of 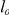 phase domains must ensue. For average domains of area 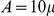
m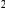 observed here, this corresponds to curvature-dependent domain ordering for all radii of curvatures below 5 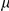
m, such as observed.

Third, an interesting corollary consequence of curvature-induced domain organization is the observed fluidity transition. Specifically, we find that the phase separated morphology of the bilayer results in the losss of long-range translational mobility of membrane molecules. The chemically distinct phases sorted by curvature produces coexisting phases with each region possessing local transition temperatures, dependent on its local molecular composition. The preferred organization of 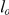 phase domains suggests that the fluid DOPC molecules concentrate in regions of higher curvature. Because DOPC is a lamellar bilayer-forming cylindrical molecule, its packing into a cylidrical motif prescribed by the patterns of high-curvature must necessarily introduce a packing frustration. [24] We surmise that this in turn reduces the long range fluidity of the entire bilayer such as observed, an interesting consequence of positional ordering of domains.

Fourth, it is notable that while the curvature-composition coupling of lipid bilayers features prominently–when coupled to real-time buckling of the underlying elastomer–it is conspicuously absent in pre-buckled membrane topographies. The distinction between these two scenarios is the dynamic wrinkling of the PDMS support under pre-formed planar lipid bilayer in the former. Our findings suggest that this dynamic membrane remodeling prompts the bilayer to change from a substrate-constrained equilibrium state, to a state in which macroscopic membrane topography and domain organization approach equilibration such as found in free membranes (e.g., vesicles and double bilayers). We propose that extraneous forces, generated at the membrane-substrate interfaces during the momentary act of buckling, provide the energy needed for the gross membrane remodeling we observe. These forces may unbind the bilayer from the substrate, or contribute energy directly to the rapid membrane reorganization.

Fifth, the rate of transformation from a flat to periodically curved substrate appears to be a key factor. In control experiments when stretched PDMS were released at slower rates (several seconds), domain reorganization is conspicuously absent. Since these samples were stretched the same amount, only the power of the release differs. Previous studies establish that the strain removal rate influences the appearance of small concentrations or defects or irregularities in the PDMS upon wrinkling, but slower or faster release of the PDMS does not affect the periodicity or morphology of the wrinkles. [12] Thus we can safely assume that the PDMS morphologies are comparable, independent of release rate. We reason that the energy associated with folding the PDMS substrate or the bilayer is dissipated before the energetic barrier is met. Unsurprisingly, no additional repartioning of domains occurs in the pre-corrugated, static control either. Reorganization is initiated when an energetic barrier is overcome, and the wrinkling mechanism in our case provides a means for delivering the necessary energy. This energetic barrier arises from strong bilayer-substrate adhesive interactions. This model can also be used to explain previous observations [11] in which reduction of the substrate-bilayer adhesion allows the distal bilayer in the double bilayer configurations to produce positional ordering of domains.

In summary, experiments reported here demonstrate how interfacial forces and curvature can couple to create conditions for mechanochemical coupling in phase-separating lipid bilayers. Phenomenologically, they appear to resemble the mechanisms that govern cardiolipin domain dynamics (pole localization and repartitioning to the septal plane) as seen in some bacteria. [9] However, this example is one of many cases of mechanochemical coupling in lipid membranes. Forces and transitory curvatures are generated *in vivo* by many disparate types of membrane-protein interactions, [25], [26] and by cytoskeletal or glycocalyx modulation. Additionally, these exogeneously-transmitted physical forces on the cellular membrane may have additional effects beyond phase separation. For instance, it is well-known that many proteins are activated by physical changes within the membrane, whether it be through forces, bending, stretching or changes in curvature, [27]–[31] even in cases where the putative role of the protein is to create or stabilize curvature in the membrane. [32], [33] We anticipate that the model systems developed here should also prove useful for studies in which the activation of such mechanosensitive proteins is desired *in vitro*.

## Materials and Methods

### 1. Preparation of PDMS

Commercially available PDMS (Sylgard Elastomer 184 kit, Dow Corning, Flint, MI) was used without further purification. The base and curing agents were mixed in 10∶1 m/m ratio. Seven grams of the mixture were poured into a 90 mm dish and allowed to sit for 5 minutes to allow bubbles to float to the surface. The PDMS is then cured at 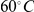 for three hours. The piece was trimmed into a square shape. This piece measures approximately 60 milimeters along the edges. The squares of PDMS were washed in a 100∶1 water and Triton-X solution, and rinsed with 18.2 m-Ohm.cm Millipore water and dried under a gentle stream of niitrogen. One pair of opposite corners was clamped to bottoms of glass dishes measuring 6 cm in diameter and 1 cm tall, and then the other corners are clamped along the orthogonal direction, so that the PDMS was flush across the bottom and there was tension along each axis. The PDMS was secured on both ends using metal binding clips. The PDMS was then placed approximately 1 cm away from a short-wavelength UV light source emitting at 185 and 254 nanometers purchased from UVP (Upland,CA). The PDMS was rinsed again with millipor water after UV exposure and was placed in an oven at 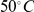 for 10 minutes.

### 2. Preparation of lipid vesicles

1,2-di-(9Z-octadecenoyl)-sn-glycero-3-phosphocholine, egg sphingomyelin (DOPC), 1,2-dilauroyl-sn-glycero-3-phosphocholine (DLPC), cholesterol, and G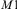 ganglioside were obtained in chloroform from Avanti Polar Lipids (Alabaster, AL). Texas Red DHPE was obtained from Invitrogen (Carlsbad, CA). A seven milliliter glass vial was rinsed with chloroform, and then the desired amounts of lipid were added to the cleaned vial. The chloroform was evaporated by rotating the vial while blowing on the chloroform with a gentle stream of nitrogen. The vial was left open and placed into a vacuum chamber for a minimum of two hours to ensure complete evaporation of the chloroform. The lipid cake was then rehydrated with millipore water to a concentration of 2 mg/ml to prepare the stock solution, and the vial was then sonicated for 15 minutes. The stock solution was stored at 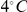 prior to use. The desired amount of hydrated aqueous solution was then sonicated prior to use, and passed through a Avanti Mini-Extruder (Avanti, Alabaster, AL) using 0.1 Î¼m polycarbonate membrane filters (Avanti, Alabaster, AL) 19 times at 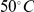. One part of the resulting SUV solutions was diluted with one part of PBS buffer and kept at 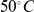.

### 3. Sample preparation and imaging

The vesicle solution was then added to a plastic dish and the PDMS was placed onto the droplets for fusion. The samples were kept in the 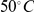 oven for 20 minutes while vesicle fusion took place. Samples were then transferred to a water bath at 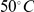 and set on a scaffold so that the bilayer did not come into contact with the bottom of the dish. After initial imaging, the samples were then placed into a bath of water at 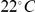 and immediately released. The sample from Fig. 3, parts e and f was released in a bath at 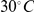. The pieces of PDMS were shaken underwater to remove excess vesicles, then trimmed to fit into a 35 millimeter plastic petri dish. The PDMS was secured to the bottom of the dish, in order to keep the PDMS from floating and to help flatten it out for observation, through the use of a binder clip attached to the side of the dish, with one of the handles pressing against the PDMS.

### 4. Treatment with Cholera Toxin subunit B

The metal clip was removed and the water in the sample dish was replaced with DPBS and 13.6 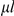 of FITC-tagged Cholera toxin subunit B, obtained from Sigma Aldrich (St. Louis, MO), assay at 0.25 mg/ml was added to the sample and left to incubate at 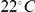 for 20 minutes. After, the sample was washed in a millipor water bath to remove excess toxin.

### 5. Fluorescence Microscopy

An inverted fluorescence microscope (Nikon, Melville, NY) was used to analyze fluorescent samples. Photobleaching was performed with high magnification objectives (40×–60×) and then recovery was monitored using a 10× objective. Software that was previously developed in our lab was used to measure the diffusion constants in the FRAP experiments. [34] The bleach spot was modeled as a time evolving Gaussian function. For more information on the mathematical modeling of this software, please consult reference 40.

## Supporting Information

Figure S1 **Epiflourescence images and data showing diffusion within the bilayer on PDMS.** In/bf a, a bleached spot on PDMS before wrinkling has occurred, and in/bf b, the corresponding curve showing the amplitude of the Gaussian spot over time. In/bf c, a bleach spot on pre-wrinkled PDMS, and in/bf d, the corresponding curve showing the amplitude of the Gaussian spot over time.
(TIF)
