# Supplementary figures and images for: Programmed Bending Reveals Dynamic Mechanochemical Coupling in Supported Lipid Bilayers
Source: PLoS One. 2011 Dec 22;6(12):e28517. doi: 10.1371/journal.pone.0028517 (PMC3245222; doi:10.1371/journal.pone.0028517)

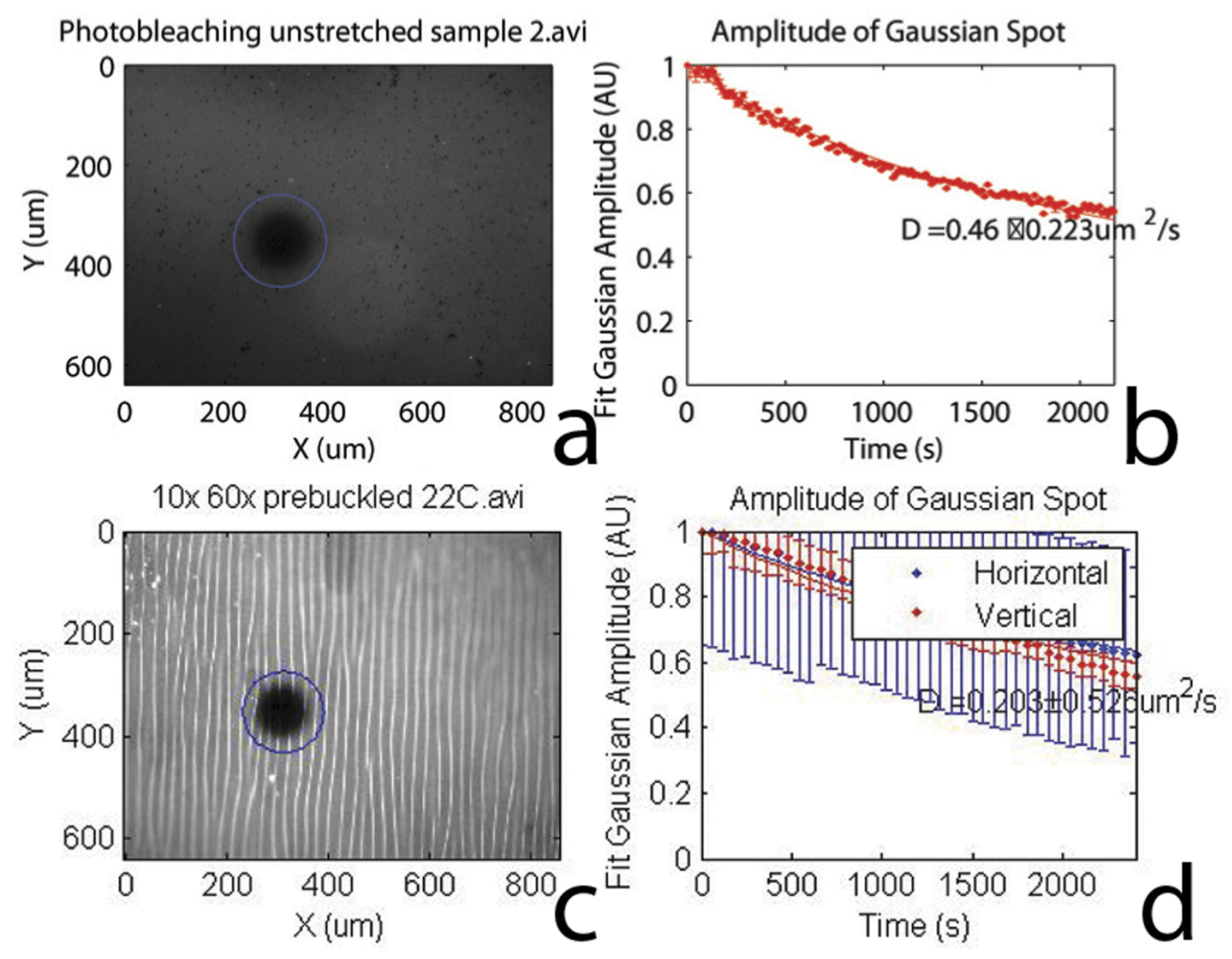

Supplement: Figure S1 — Epiflourescence images and data showing diffusion within the bilayer on PDMS. In/bf a, a bleached spot on PDMS before wrinkling has occurred, and in/bf b, the corresponding curve showing the amplitude of the Gaussian spot over time. In/bf c, a bleach spot on pre-wrinkled PDMS, and in/bf d, the corresponding curve showing the amplitude of the Gaussian spot over time. (TIF) [file pone.0028517.s001.tif]
